# The Wiring of Developing Sensory Circuits—From Patterned Spontaneous Activity to Synaptic Plasticity Mechanisms

**DOI:** 10.3389/fncir.2016.00071

**Published:** 2016-09-05

**Authors:** Alexandra H. Leighton, Christian Lohmann

**Affiliations:** Synapse and Network Development, Netherlands Institute for NeuroscienceAmsterdam, Netherlands

**Keywords:** spontaneous activity, developmental biology, visual system development, auditory system development, synaptic plasticity, plasticity mechanisms

## Abstract

In order to accurately process incoming sensory stimuli, neurons must be organized into functional networks, with both genetic and environmental factors influencing the precise arrangement of connections between cells. Teasing apart the relative contributions of molecular guidance cues, spontaneous activity and visual experience during this maturation is on-going. During development of the sensory system, the first, rough organization of connections is created by molecular factors. These connections are then modulated by the intrinsically generated activity of neurons, even before the senses have become operational. Spontaneous waves of depolarizations sweep across the nervous system, placing them in a prime position to strengthen correct connections and weaken others, shaping synapses into a useful network. A large body of work now support the idea that, rather than being a mere side-effect of the system, spontaneous activity actually contains information which readies the nervous system so that, as soon as the senses become active, sensory information can be utilized by the animal. An example is the neonatal mouse. As soon as the eyelids first open, neurons in the cortex respond to visual information without the animal having previously encountered structured sensory input (Cang et al., 2005b; Rochefort et al., 2011; Zhang et al., 2012; Ko et al., 2013). *In vivo* imaging techniques have advanced considerably, allowing observation of the natural activity in the brain of living animals down to the level of the individual synapse. New (opto)genetic methods make it possible to subtly modulate the spatio-temporal properties of activity, aiding our understanding of how these characteristics relate to the function of spontaneous activity. Such experiments have had a huge impact on our knowledge by permitting direct testing of ideas about the plasticity mechanisms at play in the intact system, opening up a provocative range of fresh questions. Here, we intend to outline the most recent descriptions of spontaneous activity patterns in rodent developing sensory areas, as well as the inferences we can make about the information content of those activity patterns and ideas about the plasticity rules that allow this activity to shape the young brain.

## Patterns of Spontaneous Activity in the Developing Sensory System

Non-evoked activity has been described throughout developing sensory systems, in visual (Torborg and Feller, 2005), auditory (Clause et al., 2014), somatosensory (Allène et al., 2008) and olfactory (Yu et al., 2004) circuits in rats and mice. It also appears across species, having been characterized in ferrets (Chapman, 2000) and cats (Godecke et al., 1997) as well as humans (Colonnese et al., 2010). The exact properties of the activity patterns can vary widely in terms of duration, spread or cell participation, and the various guises of spontaneous activity have been the topic of several excellent reviews (Blankenship and Feller, 2009; Allene and Cossart, 2010; Kerschensteiner, 2014; Kirkby et al., 2013; Ackman and Crair, 2014). Technological advances during the last decade have allowed imaging of spontaneous activity in the live animal, confirming that several types of spontaneous activity patterns exist during development in sensory regions *in vivo*.

It is possible that the different spatiotemporal properties of spontaneous activity lend themselves to performing distinct functions during development. For instance, a different type of activity may be required to produce sensory maps whilst another causes synaptic homeostasis. Though we have not yet reached a full understanding of the connection between the various types of spontaneous activity and their functions, some general patterns in the characteristics of spontaneous activity have already emerged and we can speculate on the potential consequences of their properties.

First the mechanism with which activity travels across the network can change with age. The earliest forms of spontaneous activity are often dependent on the direct exchange of electrical or chemical signals through gap-junction connections. The expression of gap-junctions between excitatory cortical cells reduces with age, until absent at P17 in the rat (Peinado et al., 1993). During this reduction, chemical synapses take over neuronal signaling. Both gap-junction mediated and chemical synaptic activity can activate specific subsets of cells, potentially creating ensembles (Yuste et al., 1992; Allène et al., 2008; Siegel et al., 2012).

Second, besides the mechanism of transfer, the size of the area activated by a spontaneous event can vary, between small local events capable of producing cortical columns (Yuste et al., 1992) or large-scale events, recruiting many cells over greater areas of the cortex (Adelsberger et al., 2005; Kirmse et al., 2015). Intuitively, the number of co-active cells have consequences for the potential function of activity, as correlated activity amongst neighboring cells in the retina or cochlea could pass on the spatial information necessary to precisely pattern sensory maps.

Around the onset of sensation, neural activity gradually changes from infrequent, high amplitude bursts, with a high number of participating cells, to an almost continuous active system with low-participation rates and reduced calcium amplitude, indicating a reduced firing rate (Rochefort et al., 2009; Siegel et al., 2012). This reduction of participation rates (Golshani et al., 2009), or “sparsification” occurs as the system shifts from a preparatory role of wiring the brain, to functional processing when the animal needs to make sense of its environment. As sparseness of encoding is associated with extracting statistics from data rather than a piece by piece replication of the input (Olshausen and Field, 2004), this could reflect the onset of visual processing and the cessation of developmental patterning (Rochefort et al., 2009). Additionally, sparse encoding is an energy efficient representation, given the smaller number of activated neurons at any one time (Olshausen and Field, 2004). Early development seems to disregard this efficiency by using high activation rates, perhaps suggesting a developmental advantage of inclusive activations despite the required energy. As the animal matures, the activity in the sensory areas gradually contains more information as activity begins to become modulated by external stimuli and vigilance state (Colonnese et al., 2010).

**Figure 1:**
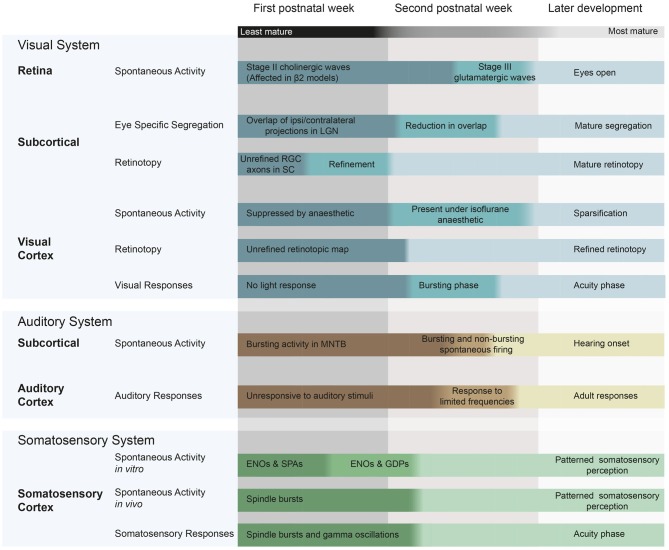
**Spontaneous and evoked activity during early postnatal development in mice and rats, and the changes in patterning that occur during this time.** Visual system: (McLaughlin et al., 2003; Cang et al., 2005a; Firth et al., 2005; Torborg and Feller, 2005; Demas et al., 2006; Rochefort et al., 2009; Colonnese et al., 2010; Siegel et al., 2012). Auditory system (Geal-Dor et al., 1993; Kandler and Gillespie, 2005; Sonntag et al., 2009; Tritsch et al., 2010; Froemke and Jones, 2011) Somatosensory system (Minlebaev et al., 2007; Allène et al., 2008; Allene and Cossart, 2010; Colonnese et al., 2010). ENO, early network oscillations; SPA, synchronized plateau assemblies; GDPs, giant depolarizing potentials; RGC, retinal ganglion cell; LGN, lateral geniculate nucleus; MNTB, medial nucleus of the trapezoid body; SC, superior colliculus.

Here, we outline current knowledge on spontaneous activity generation and propagation during the first two postnatal weeks in the visual, auditory and somatosensory systems in mice and rats (Figure 1).

### The Visual System

In rodents, the eyelids do not open until P14. Throughout these first two postnatal weeks, pacemaker cells in the retina fire spontaneously, depolarizing sequentially in waves of correlated activity that travel across the retina (as reviewed in Torborg and Feller, 2005). Activity from the retina can propagate to the superior colliculus (SC; Ackman et al., 2012) and through the lateral geniculate nucleus (LGN) of the thalamus to the primary visual cortex (V1), as the frequency of spontaneous activity in V1 drops when the eye is removed (Siegel et al., 2012). *In vivo* calcium imaging of both SC and V1 simultaneously demonstrated that the location of origin and direction of travel of the waves is matched between these areas, confirming that waves of spontaneous activity could indeed convey information about the spatial properties of the retina to the visual cortex (Ackman et al., 2012). These retinally-driven events can be identified as those in which only a subset of cells (20–80%) are active. Events in which more than 80% of cells are active are unaffected by retinal enucleation, indicating a distinct, perhaps cortical, source (Siegel et al., 2012).

During the end of the second postnatal week, the activity becomes more frequent, less correlated between cells and the amplitudes of intracellular calcium events decrease (Rochefort et al., 2009). In accordance with the imminent onset of visual input, the visual system becomes responsive to light flashes from P8. Colonnese et al. (2010) defined an early “bursting phase” in rats before eye-opening, between P8 and P11, where a bright light flash evokes a bursting pattern through the closed eyelid. During this phase, the cortical burst responses evoked by an identical stimulus vary greatly in response amplitude, the time of onset after the stimulus and the number of spikes fired. This variation in the response pattern does not depend on whether the animal is awake or sleeping. From P12, 2 days before eye opening, the next phase begins where cortical responses to a stimulus become consistent and these responses are modulated by the vigilance state of the animal.

### The Auditory System

In the cochlea, inner hair cells show spontaneously generated calcium action potentials before hearing onset. The initiation of these action potentials is triggered by glia-like inner supporting cells through a remarkable mechanism. These cells release ATP, activating a flow of chloride out of supporting cells through TMEM16A channels. Water and potassium follow the chloride efflux, leaving the supporting cell and causing transient osmotic shrinkage. The high extracellular potassium depolarizes inner hair cells (Wang et al., 2015), triggering glutamate release and producing bursts of action potentials in spiral ganglion cells. At this synapse, NMDA receptors act as an amplification mechanism, prolonging post-synaptic currents and enhancing depolarizations in spiral ganglion cells, enabling their fast spontaneous firing rate. This NMDA-dependent increased excitability also controls how many cells are activated by each inner hair cell depolarization, as pharmacologically blocking NMDA receptors reduces the number of spiral ganglion cells that participate in each event (Zhang-Hooks et al., 2016). Such peripherally generated activity can propagate via the auditory nerve to the rest of the auditory system, as action potentials in the medial nucleus of the trapezoid body (MNTB) and the inferior colliculus were abolished by removal of the cochlea (Tritsch et al., 2010). For an extensive overview of spontaneous activity in the developing auditory system see Wang and Bergles (2015).

Before P11, a combination of various mechanical factors prevent hearing, though bone conducted stimuli cause an auditory brainstem response from P7 (Geal-Dor et al., 1993). The brainstem tonotopic map can respond to the mature range of frequencies by P14 (Friauf, 1992). The auditory cortex also develops quickly at P10, the cortex does not respond to tone stimuli. At P11, A1 responds only to high-intensity stimuli between 6 and 10 KHz (de Villers-Sidani et al., 2007), and it reaches the adult level of responsiveness at P14. During this time, A1 greatly increases in size and represents an increasing range of frequencies and intensities. For a review of this development, see Froemke and Jones (2011).

### The Somatosensory System

The somatosensory system matures earlier than the visual and auditory systems in rodents. The onset of both hearing and sight occurs after birth, allowing postnatal *in vivo* experiments during which measured activity cannot be directly evoked though external stimuli and therefore can be classified as spontaneous. In contrast, the somatosensory cortex responds to sensory stimulations from P2, creating some difficulty when measuring its spontaneous activity *in vivo*—particularly in awake animals where somatosensory inputs cannot be fully prevented. After P8, the somatosensory cortex enters the “acuity phase” where stimuli cause reliable responses (Colonnese et al., 2010).

In cultured slices of the somatosensory cortex, both gap junction mediated and synaptic forms of spontaneous activity have been described, including gap-junction mediated synchronized calcium plateau assemblies, NMDA dependent early network oscillations (ENO) and GABAergic giant depolarizing potentials (Allene and Cossart, 2010). Similar activity patterns occur in the somatosensory cortex *in vivo*. There, early gamma oscillations (EGOs) can be measured during the first postnatal week. These short oscillations typically remain within one cortical barrel and rely on rhythmic input from the thalamus (for a review on EGOs see Khazipov et al., 2013). Together with spindle bursts of around 10 Hz, measured between P0 and P8 *in vivo* (Khazipov et al., 2004; Yang et al., 2009), EGOs form a young sensory response during the first postnatal week. This underlines the precocious maturation of the somatosensory cortex in comparison to the visual and auditory cortices, though spindle bursts can occur in the absence of sensory inputs (Khazipov et al., 2004).

## Spontaneous Activity Helps Wire the Developing Sensory System

The above descriptions paint a picture of spontaneous activity as a pervasive phenomenon during development, but do not directly demonstrate its function. Blocking spontaneous activity often causes severe disruptions of the organization of sensory areas, indicating its importance for precise wiring of the developing brain (Cang et al., 2005b; Chandrasekaran et al., 2005). This importance is underlined by the observation that spontaneous activity patterns are different in neurodevelopmental disorders such as Fragile X syndrome (Gonçalves et al., 2013).

Since early experiments in which spontaneous activity was eliminated, our grasp on the rules which underlie the patterning of the neonatal brain has greatly increased. This is mainly due to technical improvements which have allowed subtle and specific manipulations of spontaneous activity rather than overall elimination. These exciting experiments aimed to clarify the information content of these waves and which characteristics (for instance, their timing, spatial properties, frequencies or wave amplitudes) are important for their function. Thanks to this recent work, we can begin to sketch out the role of spontaneous activity, more clearly delineating which processes do and which do not rely on intrinsically generated activity.

### The Visual System

As described above, one major source of spontaneous activity in the visual system is the retina. The wave characteristic of spontaneous activity in the developing retina provides spatiotemporal information—as the wave travels, neighboring retinal ganglion cells (RGCs) will fire in turn, passing on information about their spatial relationship in the temporal properties of the wave. When a mouse opens its eyes at around P14, neurons in the V1 have already been thoroughly organized according to the spatial structure of the retina; V1 shows retinotopic maps, eye-specific segregation and orientation tuning of individual neurons (Smith and Trachtenberg, 2007; Rochefort et al., 2011; Ko et al., 2013).

In the visual system, RGCs representing adjacent parts of the visual field project to neighboring cells in the visual cortex, resulting in a retinotopic map. Initially, the retinotopic maps set out in the visual system are instructed by molecular guidance cues, such as ephrins- the ligands of the Eph receptor tyrosine kinases, which guide projections from the LGN to V1 (Cang et al., 2005a). These cues appear in a gradient across the target area, giving some directional information that results in a coarse retinotopic organization. This initial targeting is activity independent (Benjumeda et al., 2013). Futher fine-tuning of these connections, then occurs through both pruning of excessive connections and increasing arborization within the correct termination zones (Simon and O’Leary, 1992). Anatomically, refinement is measured through labeling of projections and determining the size of the area in which they terminate. This refinement can also be measured functionally, by determining the size of the regions within the higher visual areas that are activated by a given stimuli. It is important to study refinement at both the single cell an population level, as a population that represents a large part of the visual field can be made up of many neurons that are each broadly-tuned, or a group of individually finely-tuned neurons with large variation in receptive fields (Mrsic-Flogel et al., 2005).

### Retinotopy and Eye Specific Segregation

One of the most common models of disrupted retinal activity is the β2 global knock-out mouse. These mice lack the β2 subunit of the nicotinic acetylcholine receptor. They have altered retinal activity during the first postnatal week and in turn, disrupted patterning of higher visual areas. In contrast to wild-type, in which a clear wave-front travels over the retina in a successive activation of ganglionic cells, the β2 global knock-out shows almost simultaneous activation of much larger groups of neurons. These waves are gap-junction dependent and of lower frequency and amplitude when compared to controls. When dye injections are used to visualize geniculocortical projections, the termination areas are larger in β2 knock-out mice than in wild-type (Cang et al., 2005b), and this corresponds to less fine-tuned functional retinotopy in the SC (Mrsic-Flogel et al., 2005), LGN and V1 (Grubb et al., 2003; McLaughlin et al., 2003; Cang et al., 2005b). Additionally, the segregation of terminals from the ipsilateral and contralateral eyes (eye-specific segregation) is disrupted. The propagation of activity through the visual system also seems to be changed, as the SC shows “extra”, short waves that do not correspond to retinal activity (Burbridge et al., 2014).

#### Retinotopic Patterning Requires Locally Correlated Activity

**Figure 2:**
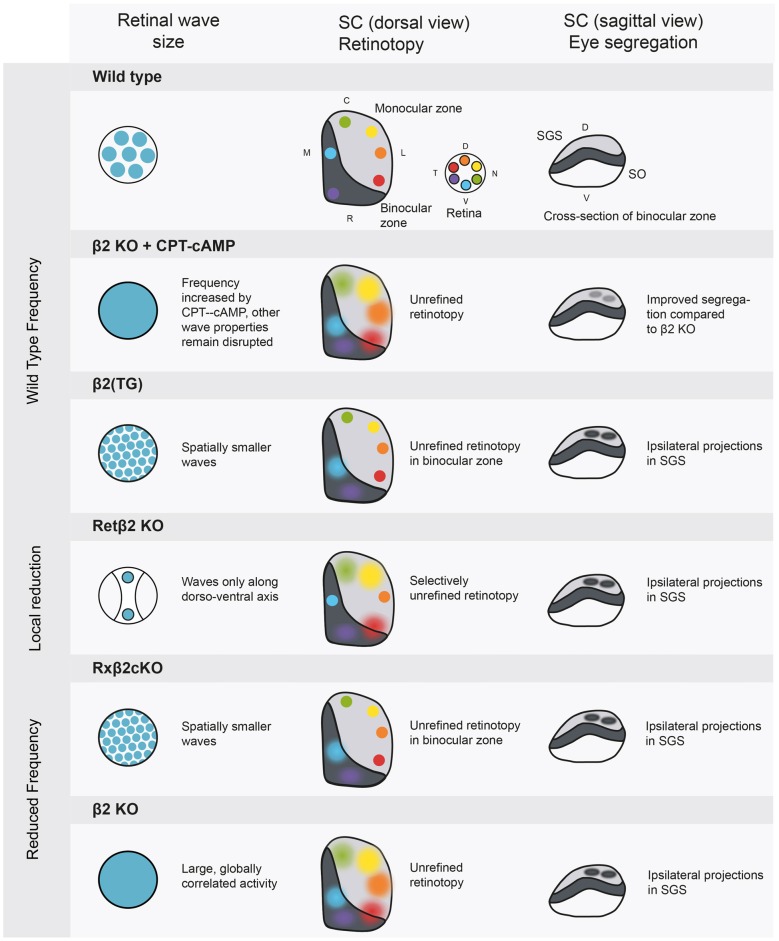
**Manipulations of spontaneous activity frequency and wave size and the consequences for retinotopy and eye-specific segregation in the SC.** The SC has a binocular and a monocular region, and contains a retinotopic map in a mirror image of the retina. The most superficial layer of the SC is the stratum griseum superficial (SGS), which is targeted only by axons from the contralateral eye. The stratum opticum (SO) contains ipsilateral projections in wild type animals. *Wild type*: retinal activity in the wild type mouse. β2 knockout+ cAMP-CPT: this manipulation increases frequency to wild type levels (Burbridge et al., 2014), rescuing eye specific segregation but not retinotopy. β2 (TG): truncated waves as in the β2 (TG) mouse disturb eye-specific segregation (Xu et al., 2011). Retβ2-KO: partially disrupting wave activity also has spatially selective consequences for retinotopy (Burbridge et al., 2014). Rxβ2-KO: reducing wave frequency and size disturbs segregation (Xu et al., 2015) and retinotopy in the binocular zone. β2 KO: the whole body β2 knockout has low frequency activity over large areas of the retina, leading to unrefined retinotopy and disrupted eye specific segregation. SGS, stratum griseum superficiale; SO, stratum opticum; D, dorsal; V, ventral; C, caudal; R, rostral; T, temporal; N, nasal.

One major limitation of the β2 knock-out mouse is that both the frequency and the spatial properties of retinal activity are different to wild-type mice (Figure 2), making it difficult to tease apart the relative influence of each factor on the phenotype. Several variations of the β2 mouse have been used to more specifically manipulate activity patterns by varying either the frequency or the spatial spread of waves (for an excellent review of the consequences of many of these disruptions see Kirkby et al., 2013). These experiments have allowed us to link specific properties of spontaneous activity to higher area patterning. The first conclusion we can begin to draw is that retinotopy seems to depend on local correlations within retinal waves and not on firing rate. Retinotopy is disrupted in mouse models with a large retinal wave, where large areas of the retina become correlated. In contrast, genetic models in which local correlations between neighboring cells are maintained consistently have accurate retinotopy (Xu et al., 2011, 2015). The mutant mouse Rx-β2cKO has a lower than wild-type firing frequency, but shows normal retinotopy and restoring the firing rate of retinal waves in the β2 knock-out to wild-type levels does not save the retinotopic map (Burbridge et al., 2014; Figure 2).

Recently, Burbridge et al. (2014) used a Ret β2-cKO mouse (Figure 2) as an elegant demonstration of the link between retinal waves and higher area retinotopy. In this mouse, the β2 subunit is knocked out selectively in temporal and nasal areas of the retina, locally canceling wave activity during stage II cholinergic waves. The rest of the retina, along the dorso-ventral axis, showed clearly propagating waves. This is reflected in the SC at P8, where terminations from the altered, naso-temporal retina spread over a larger area than those from the non-expressing areas. Overall, it seems that retinal waves contain local spatial information about the retina essential for normal retinotopy, indicating an instructive role for spontaneous activity. However, Zhang et al. (2012) debate the role of spatial properties in retinotopic mapping. They used channelrhodopsin in RGCs to create artificial retinal waves in which all simulated cells fired simultaneously, removing the local spatial information. They report very little effect of this stimulation on contralateral axon retinotopy in the SC. However, as the stimulation occurred at P9, it is likely that some retinotopy had been set up before the time of stimulation. A learning rule put forward by Butts et al. (2007) postulates that the initial strength of connections will bias subsequent activity competition in favor of the more strongly connected wiring, perhaps explaining why retinotopy was not reorganized by synchronous stimulation occurring after the circuit was established. Indeed, stronger stimulation may have strengthened these connections, reinforcing the existing map (Kirkby et al., 2013).

#### Eye-Specific Segregation Depends on Firing Frequency and Inter-Eye Synchronicity

In both the LGN and SC, projections from both eyes initially terminate in partially overlapping areas (Demas et al., 2006). During the first two postnatal weeks, these terminations are refined, clearly dividing where inputs from each eye are segregated and where they are combined to produce binocular vision. In contrast to retinotopy, this eye-specific segregation does depend on the firing rate of spontaneous activity, as restoring the firing rate of the retina in whole body β2 knock-out mice using CPT-cAMP improves segregation (Burbridge et al., 2014). Results from the Rx-β2cKO mouse confirm this, as their lower frequency of relatively normal activity results in selective disruption of eye-specific segregation (Xu et al., 2015), Figure 2. Overall firing rate cannot be the only important factor, as mice with wild-type frequency, but spatially smaller waves (β2 (TG)) have disrupted eye-specific segregation (Xu et al., 2011). It therefore seems that spatial information is important, but at a different scale than for retinotopy; rather than local correlations amongst neighboring cells, the overall area activated by each wave may be essential in eye-specific segregation. There may be an activity threshold for segregation, which can either be reached by frequent or by large-scale activity.

Zhang et al. (2012) also used their protocol for optogenetic activation of RGCs to test the role of spontaneous activity in eye-specific segregation of the SC and LGN. The more the stimuli overlapped between the eyes, the more the disruption in eye-specific segregation worsened. Synchronous stimulation could also disrupt segregation even after eye-specific segregation, indicating an important role for retinal waves not only in creating but also in maintaining segregation, as has been previously reported (Demas et al., 2006). Asynchronous stimulation of the eyes (with more than 100 ms difference) did not disrupt segregation, suggesting a sub-second resolution of this competition. It is usually assumed that, as retinal waves arise spontaneously, retinal or SC waves from both eyes are not synchronized. However, Ackman et al. (2012) found that 15% of retinal waves are temporally matched between the eyes and proposed that descending synchronized inputs or synaptic interaction between the eyes mediated this synchronicity. As mentioned above, Burbridge et al. (2014) report that the β2 knock-out mouse has a much higher correlation of wave activity between the two retinas, which could contribute to the reduced segregation seen in this model.

The retina has different stages of wave activity, starting with gap-junction coupled stage I waves and maturing to cholinergic stage II waves from P0. From P11, glutamatergic stage III waves take over (Firth et al., 2005). The retinal wave stages have different activity properties, the importance of which is not fully understood. Xu et al. (2016) report that when stage II waves are disrupted, eye-specific segregation is affected. This disruption can be rescued by a period of stage III waves. However, when stage II waves persist throughout the second postnatal week (i.e., the system does not transition into stage III glutamatergic waves) it does not affect segregation in the LGN or SC (Xu et al., 2016). It seems that stage II waves are important for segregation, and that activity during the second postnatal week (but not specifically the properties of stage III waves) can still influence this segregation.

The difference between retinotopy and eye-specific segregation is not a complete opposition; the sensory system must be able to form and maintain both patterns simultaneously and problems with one can interrupt with the other. Excellent examples of this occur in the Rx-β2cKO and β2(TG) mice models, which both have normal retinotopy in monocular regions, but disrupted retinotopic mapping in binocular regions of the SC. It seems that the localized problems with retinotopy are a consequence of problematic eye-specific segregation. The strongest evidence for this, is the observation that when one eye is removed at birth, therefore removing inter-eye competition, all retinotopy from the remaining eye stayed intact in both the SC and LGN (Xu et al., 2011, 2015). When both eyes are present, but eye-specific segregation does not occur, the unpruned ipsilateral projections convey out-of-sync spontaneous activity, that may disrupt the retinotopic map.

### Direction and Orientation Selectivity

Some RGCs respond specifically to one of four cardinal directions. These RGCs are contacted by starburst amacrine cells whose synapses are, at first, uniformly present over the dendrites. During the second postnatal week, direction selectivity emerges as GABAergic inhibitory current increases on the side of the cell opposite to the preferred direction, likely due to a selective increase of synaptic strength (Wei et al., 2010). The development of direction selectivity of the retinal cells occurs independently of spontaneous activity, as intraocular injections of muscimol or gabazine (selective GABA-A receptor agonist and antagonist, respectively), administered between P6 and P12, did not alter this developmental trajectory (Wei et al., 2010).

Despite not being required for setting up the direction selectivity in the retina, spontaneous activity traversing the system could contain the information needed to pattern the cortex according to RGC direction selectivity. Additionally, in mouse V1, neurons are tuned to direction from eye-opening. This early selectivity is independent of any visual experience as it is not prevented by dark-rearing (Rochefort et al., 2011), suggesting that spontaneous activity may mediate orientation and direction selectivity. Surprisingly, a recent study found that blocking spontaneous activity during development in mice did not reduce orientation selectivity (Hagihara et al., 2015). Spontaneous activity was blocked in L2/3 of the visual cortex, by expressing the inward rectifying potassium channel Kir 2.1 through *in utero* electroporation. Expression of Kir 2.1 in only a small subset of L2/3 neurons (4.6% in central V1, through to 30% in anterior V1) was enough to significantly reduce synchronized activity in both L2/3 and L4. Surprisingly, when the visual responses of these neurons were measured in adult mice, they were equally responsive and selective to visual stimuli with different orientations as neurons in the control animals. This also occurred when animals were reared in darkness. The role of spontaneous activity may vary per brain region, as SC cells in the β2 knock-out mouse have reduced orientation and direction selectivity (Wang et al., 2009).

### The Role of Spontaneous Activity in Visual System Patterning Varies Between Species

Much of the work done in spontaneous activity has focused on rats and mice, but it is vital to note the important experiments carried out *in vivo* in the ferret. Many of the same patterning processes as in the mouse also take place in neonatal ferrets, which open their eyes after the fourth postnatal week. However, the significant spatiotemporal properties and the permissive or instructive nature of spontaneous activity may vary between species. Spontaneous activity is clearly important in the ferret visual system, as it is able to drive refinement. When Davis et al. (2015) pharmacologically increased the frequency of spontaneous activity between P15 and P25, at the stage of glutamatergic retinal waves, they were able to actually accelerate the normal refinement of LGN receptive fields. After eye opening, the animals with more retinal waves had smaller receptive fields than saline controls.

One clear difference between the ferret and mouse is in eye-specific segregation. Ablating starburst amacrine cells in the ferret retina reduced the size of retinal waves and inter-cell correlations, but did not prevent normal eye-specific segregation (Speer et al., 2014). This is in contrast to the mouse, where smaller retinal waves did disrupt the accurate segregation of ipsi- and contralateral projections, although it is not straightforward to directly compare wave size between species.

In the ferret, neurons responding to orientation have been found from P23, before eye opening. During the following 3 weeks, more neurons become responsive to the orientation and the average selectivity of the responsive population increases (Chapman and Stryker, 1993). This maturation depends on neuronal activity; orientation tuning at 6 weeks old was somewhat reduced after artificially correlated activity was produced through stimulation of the optic nerve (Weliky and Katz, 1997). If activity is silenced through TTX application between postnatal weeks 4 and 7, orientation tuning fails to mature beyond the level found at 4 weeks of age. Preventing visual experience through binocular eyelid suturing also greatly impairs the development of orientation selectivity, but not to the same extent as when all activity is blocked (Chapman and Stryker, 1993), suggesting a role for spontaneous activity. Cells in the ferret V1 are not direction selective at eye opening, but become selectively responsive to direction over the next few days. Importantly, visual experience is required for this maturation (Li et al., 2006). Seemingly in contrast, in the mouse, Hagihara et al. (2015) found that the proportion of responsive and orientation-selective cells is mature at eye opening. This is in line with findings from Ko et al. (2013) but unlike Rochefort et al. (2011) where a gradual increase in responsiveness and selectivity was reported. At eye opening, there is a bias for cells to respond to lines with a 90° angle (Rochefort et al., 2011; Hagihara et al., 2015). This bias is equalized after eye opening, which depends on activity but, critically, does not require visual experience (Hagihara et al., 2015). It seems that the development of direction and orientation tuning is challenging to directly compare between the ferret and mouse. One possible cause for these discrepancies is a different developmental time course; the effect of manipulations such as TTX administration could depend greatly on how much patterning has already occurred. The underlying question is whether the same developmental processes occur in these animals. Though it seems likely that the computation of orientation selectivity is comparable across species (Kondo and Ohki, 2016), the differences in the organization (mice, for instance, lack orientation columns) may lead to different requirements when wiring up the brain.

### Auditory System

There are very clear parallels between the development of the visual and auditory system, such as the need for maps (tonotopic or retinotopic) and the combination and segregation of inputs from the left and right sensory organs. Throughout the auditory system, a tonotopic map is maintained, organizing projections depending on the sound frequency they represent.

In terms of spatiotemporal characteristics of spontaneous activity, auditory activity may contain equivalent information to visual retinal waves; not only are they grouped into bursts that synchronize tonotopically similar cells (Kandler et al., 2009), in chick embryos these bursts also contain information about the frequency sensitivity of the hair cells (Lippe, 1995).

Information from the cochlea enters the ventral cochlear nucleus via the auditory nerve. Subcortically, the lateral superior olive (LSO) encodes the inter-aural sound amplitude differences, required for auditory localization, by receiving excitatory inputs from the ipsilateral ear and inhibitory inputs from the contralateral ear via the MNTB (Kandler and Gillespie, 2005). To identify the important properties of spontaneous activity in the subcortical auditory system, Clause et al. (2014) worked with a mouse model in which the α9 subunit of the Nicotinic acetylcholine (nAch) receptor was knocked out. The inner hair cells are transiently innervated by cholinergic fibers from the medial olivocochlear bundle (for a discussion of transient synaptic connections in spontaneous activity during development, see Blankenship and Feller, 2009). When the α9 subunit is knocked out, this results in bursts of activity at the same overall firing frequency, but organized into shorter bursts with more action potentials per individual burst. Deleting the α9 subunit led to less refined tonotopy—the LSO was targeted by a larger region of the MNTB, and received many more connections. The overall amount of inhibition received was the same, as each connection had weaker synapses than in wild type. It is striking that a subtle change in the temporal properties of cochlear spontaneous activity, whilst preserving the overall firing rate, led to such disruption of developmental organization. This result suggests that the auditory system, similar to the visual system, is sensitive to the information content of spontaneous activity.

In contrast to subcortical areas, the auditory cortex is very immature at the time of hearing onset (P11). At this time, only a small area of the auditory cortex shows tuned responses, selectively to frequencies of around 7 Hz. Besides tuning, the latency between stimulus onset and cortical response is longer than in the adult. The tonotopic map matures quickly, reaching its adult size and mapping at P13-P14, whereas response latencies take longer to mature (Froemke and Jones, 2011). As A1 shows little patterning before hearing onset, this may indicate that spontaneous activity has a relatively small role in auditory cortical development when compared to the auditory brainstem or visual cortex.

## Plasticity Mechanisms in Spontaneous Activity

The above studies describe changes in development caused by spontaneous activity. We do not yet fully understand the mechanisms that guide these changes—which electrical signals, chemical factors and plasticity rules determine how altered temporal or spatial patterns can change the organization of the network. For instance, we do not have a clear idea of the signals received by a cell that cause the synaptic elimination during retinotopic refinement. Only by directly observing the plasticity mechanisms at work at these synapses can we really link the information content of the activity to the structural and functional changes it causes.

Different plasticity mechanisms exist and may function side-by-side. Both refinement and homeostasis occur in many manipulations of activity which result in unrefined axonal projections—these neurons have larger termination areas, made up of more individual fibers than in wild type. When these projections are functionally tested, the overall innervation strength is similar, as each individual axon has a weaker effect on the postsynaptic cells (Clause et al., 2014; Lee et al., 2014). This suggests an interesting homeostatic plasticity mechanism—a system is in place to ensure that the overall projection strength is maintained. A similar pattern of many, weaker synapses is found in the auditory brainstem of the Cav1.3 knock-out mouse (Hirtz et al., 2012).

### Long-Term Potentiation (LTP) and Long Term Depression (LTD)

Classically, the Hebbian postulate that neurons that fire together and wire together has been thought to underlie the developmental shaping of the higher areas by peripherally generated spontaneous activity. The repeated firing of presynaptic neurons together with the cells to which they project, strengthen those feedforward connections. In addition, lateral connections between postsynaptic cells that are simultaneously depolarize become potentiated. Conversely, when a postsynaptic cell fires an action potential without presynaptic glutamate release the strength of the connection decreases—therefore an existing strong connection can indirectly decrease the strength of other inputs by causing asynchronous action potentials.

**Figure 3:**
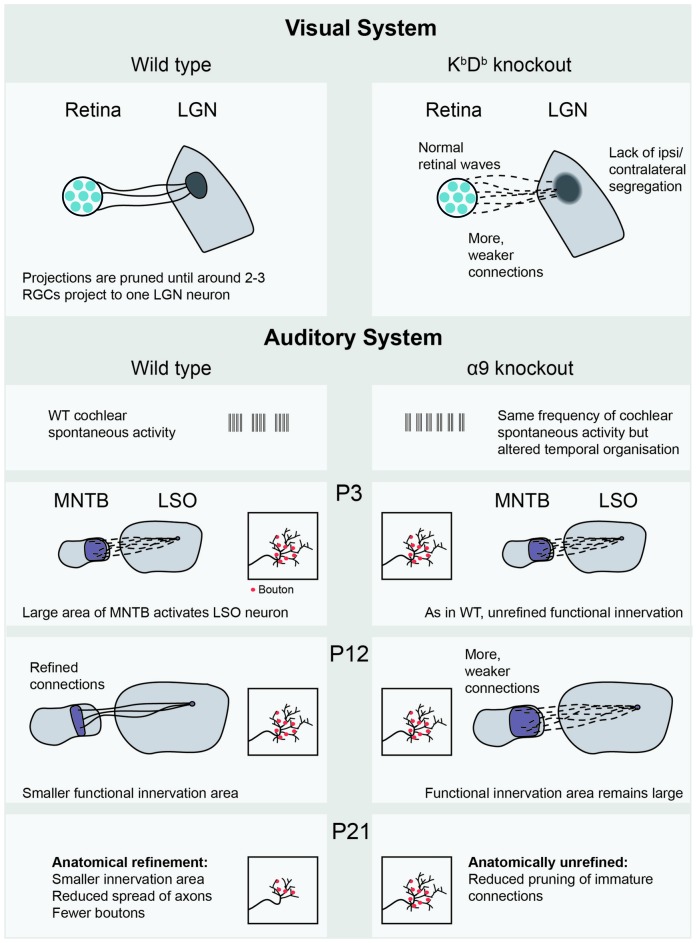
**Recent empirical evidence for Hebbian and homeostatic plasticity mechanisms mediated by spontaneous activity.** The K^b^D^b−/−^ knockout (Lee et al., 2014) shows lack of LTD and altered retinogeniculate projections, in which many weak projections connect the retina to the LGN. In the auditory system, the MNTB-LSO projection in the α9 subunit knockout (Clause et al., 2014) shows both functional and anatomical consequences of altered cochlear spontaneous activity, occurring at different postnatal ages. LGN, Lateral geniculate nucleus; RGC, retinal ganglion cell; WT, wild-type; MNTB, medial nucleus of the trapezoid body; LSO, lateral superior olive; P3, postnatal day 3.

There is empirical evidence for Hebbian learning during sensory development. Žiburkus et al. (2009) used 50 Hz spike trains in bursts of 1 s to mimic retinal activity. This stimulus could induce LTD at the rat retinogeniculate synapse *in vitro*, but only up until P14, after which the same protocol induced synaptic potentiation. Lee et al. (2014) examined the same synapse in mice, using a model with altered AMPA receptors. This mouse lacks the major histocompatibility complex (MHC) class 1 immune proteins H2-K^b^ and H2-D^b^ (K^b^D^b−/−^), which does not affect their retinal waves but does impair eye-specific segregation in the thalamus (Figure 3). In healthy animals, the convergence from the retina to the LGN is developmentally reduced until only 1–3 RGCs project to each postsynaptic cell, but the K^b^D^b−/−^mouse does not show this reduction in projection number. In these animals, LTP could be induced normally, through pairing LGN cell depolarization with a presynaptic 10 Hz activity train. However, when pre- and postsynaptic stimulations were offset in time in an attempt to cause LTD, the synapses did not weaken. This imbalance towards LTP was due to the high calcium permeability of the AMPA receptors in the K^b^D^b−/−^ mouse—when their permeability was reduced, LTD could be induced. Restoring H2-D^b^ only to neurons rescued the phenotype, indicating that the protein specifically plays a role in neurons rather than in a systemic immune response. Interestingly, the overall amount of excitation received by each postsynaptic cell was the same—the higher number of terminating fibers was compensated for by each fiber having a weaker synaptic strength, implying that there is a homeostatic mechanism at work. These results suggest a strong link between the ability for synapses to weaken through LTD and the removal of excessive axons.

Similar refinement is necessary in the auditory system. As the LSO grows in size between P4 and P12, the projecting axons from the MNTB expand to compensate for this, maintaining a stable projection size. This growth also occurred in α9 knockout animals, where the temporal properties of cochlear spontaneous activity were altered (Clause et al., 2014). At P12, measurements of bouton spread over the tonotopic axis were made, an anatomical measurement that reflects how much of the tonotopic map the observed neuron can innervate. These measurements were indistinguishable from wild-type (Figure 3). Functional recordings, however, showed some differences. The total strength of the whole projection was the same, but was made up of more, individually weaker connections in the α9 knockout, reminiscent of the homeostasis reported in the K^b^D^b−/−^mouse (Lee et al., 2014). The apparent contradiction of unrefined functional, but refined anatomical measurements, might be explained through the existence of silent synapses in the wild type animal. These synapses would show up in anatomical measurements of boutons, but not in functional analyses. In both WT and α9 knockout animals, new boutons were selectively added to the center of tonotopic receptive fields even before hearing onset. After hearing begins, spatially uniform synaptic pruning takes place, expressively described by the authors as a “sinking iceberg” model. This form of refinement also did not occur in the α9 knockout, resulting in less specific anatomical and functional innervation at P21. This suggests that spontaneous activity plays a role in both (un)silencing synapses and anatomical pruning, though not necessarily at the same time. These extensive changes in functional and anatomical refinement are particularly fascinating given that the change in spontaneous activity was relatively minor: the overall activity level was the same, but each burst (which occurred more frequently) was shorter. Typically, functional differences in synaptic strength are quickly converted into structural changes such as bouton elimination. However, the time offset between functional and structural refinement suggests that this could be an excellent model for separating synaptic plasticity mechanisms. It seems that functional synaptic strengthening and anatomical elimination are not always a package deal, and that different rules and mechanisms underlie each phenomenon. The use of the α9 knockout has opened up a new way of investigating auditory development, raising many new questions. For instance, the MNTB-LSO projection is inhibitory, which may affect the plasticity mechanisms at work. To strengthen the link between this model and the extensive literature in the visual system, it will be interesting to see how much of the tonotopic map is activated by waves in the α9, perhaps allowing the comparison with retinal wave size in the β2 variants.

LTD and LTP learning could build up some aspects of the maps necessary for sensory processing. However, there are some results that cannot be explained by classic Hebbian learning, clearly set out in Kirkby et al. (2013). Additionally, synaptic organization is observed at a subcellular level (see below), which cannot directly be explained through this mechanism. In order to really understand the rules that work together to build young brains, we need a more thorough understanding of the various types of plasticity mechanisms.

### Gap Junctions and Connection Specificity

Early in development, network activity relies heavily on coupling through gap junctions, which permits the creation of assemblies (Yuste et al., 1992) that can become active simultaneously (Kandler and Katz, 1998), for review see Niculescu and Lohmann (2013). Because their cytoplasm is directly linked, neurons can share electrical signals and exchange small molecules (Shimizu and Stopfer, 2013). As the animal develops, gap junctions disappear and signaling is fully taken over by mature chemical synapses, through which cells signal to each other using neurotransmitters. It is not yet known how the network shifts between these two types of connections. Important studies concerning this change have been focused on clonally related cells, which are neurons that originate from divisions of the same precursor cell. Not only are these clones more likely to be connected by gap junctions than non-clonally related cells during postnatal days 1–6 (P1–6), they are also more likely to form chemical synapses in animals from P9 (Yu et al., 2009, 2012), and this preference relies on gap junctions (Yu et al., 2012). It is possible that the repeated correlated firing of cells during spontaneous activity maintains the pattern of cell connections set up by gap junction coupling whilst the network changes to rely on chemical synapses. A recent article modeled a potential link between early gap junction connections and later chemical synapses (Ko et al., 2013). In this model, the electrical coupling provided by gap junctions increased the likelihood that linked cells would fire action potentials simultaneously. Because of this co-activity, clonally related cells were more likely to fire in response to the same set of feedforward inputs, stabilizing the same presynaptic connections according to the Hebbian postulate. Given that clonally related cells show similar orientation preferences (Li et al., 2012; Ohtsuki et al., 2012), these findings may have significant consequences for our understanding of visual development. It is important to consider that blocking spontaneous activity during the age at which the system transitions from gap-junction to chemical synapse signaling did not prevent normal orientation tuning (Hagihara et al., 2015). Together, these studies emphasize the importance of future studies to outline where spontaneous activity is, and is not, necessary for sensory development.

### Dendritic Organization

In recent years, there has been a surge in our understanding of the computational power of a neuron. The classic description of a neuron is as a linear integrator, summing inputs evenly regardless of their position along the dendritic tree. Recently, experimental evidence has come to support the idea that dendritic compartments can act as computational units, integrating inputs in a non-linear fashion (Poirazi and Mel, 2001), for review see Govindarajan et al. (2006); Larkum and Nevian (2008); Branco and Häusser (2010) and Winnubst and Lohmann (2012). Spatially clustered synapses can exert increased influence on cell output when they are simultaneously active by generating NMDA dependent “dendritic spikes”—large events whose charge exceeds the linear summation of the synapses involved. For this to have functional advantages, strategic organization of synapses along the dendrite is required. Such dendritic specificity and the implications for the output of the cell has been demonstrated in adults (Lavzin et al., 2012; Sheffield and Dombeck, 2015).

During spontaneous activity in development, synapses along the dendrite that are closer together (<12 μm) are more likely to be active simultaneously. This organization disappears quickly when spontaneous activity is blocked (Kleindienst et al., 2011). An “out of sync, lose your link” plasticity rule underlies this organization, as synapses that show low synchronicity to their neighbors become depressed through a significantly decreased transmission efficiency (Winnubst et al., 2015). It is essential to understand how these changes, induced by activity patterns, are signaled to individual synapses. It seems likely that proBDNF, acting on the p75^NTR^ receptor, acts as a local “punishment factor” for synapses with low co-activity levels (Winnubst et al., 2015).

Spatial clustering was found in both the visual cortex *in vivo* and the hippocampus *in vitro*. Though there are many similarities between the mechanisms of clustering these two areas, they have different temporal characteristics—a burst in the hippocampus lasts only around 400 ms, whereas bursts in the visual cortex have a longer duration of around 2 s. Interestingly, this was reflected in the plasticity rules guiding synaptic depression. When probing the time window during which two synapses were considered coactive, the hippocampus showed a much shorter integration window, of 400 ms, whereas in V1, depression of synapses was prevented if they were coactive within 2 s. Burst duration could be an important property of spontaneous activity, linking together only cells that are active within a certain time window.

It is possible that clustering of synchronized inputs and similar new plasticity rules could work together with Hebbian mechanisms to provide a range of options for patterning the developing brain. For instance, the depression of out-of-sync synapses is a tempting rule to apply to eye-specific segregation, where competition-based elimination takes place. However, we have little empirical evidence of how this might occur. As current techniques now allow us to directly measure changes at the level of the “nuts and bolts” of the developing brain, we are set to begin to really understand the rules according to which the nervous system is built.

## Author Contributions

AHL and CL wrote the manuscript.

## Conflict of Interest Statement

The authors declare that the research was conducted in the absence of any commercial or financial relationships that could be construed as a potential conflict of interest.
